# Filling the gaps: ethnobotanical study of the Garrigues district, an arid zone in Catalonia (NE Iberian Peninsula)

**DOI:** 10.1186/s13002-020-00386-0

**Published:** 2020-06-09

**Authors:** Airy Gras, Joan Vallès, Teresa Garnatje

**Affiliations:** 1 Institut Botànic de Barcelona (IBB; CSIC-Ajuntament de Barcelona), Passeig del Migdia s.n., Parc de Montjuïc, 08038 Barcelona, Catalonia Spain; 2 Laboratori de Botànica (UB) - Unitat associada al CSIC, Facultat de Farmàcia i Ciències de l’Alimentació, Institut de Recerca de la Biodiversitat – IRBio, Universitat de Barcelona, Av. Joan XXIII 27-31, 08028 Barcelona, Catalonia Spain; 3 Institut d’Estudis Catalans, C. del Carme 47, 08001 Barcelona, Catalonia Spain

**Keywords:** Arid zones, Catalonia, Ethnobotany, Ethnopharmacology, Food uses, Garrigues, Iberian Peninsula, Medicinal uses, Plant uses

## Abstract

**Background:**

This study has focused on the Garrigues district, one of the most arid regions in Catalonia (NE Iberian Peninsula), which, in general terms, has remained unexplored from the ethnobotanical point of view. This area, of 22,243 inhabitants, comprises 33 municipalities distributed across 1123.12 km^2^. The natural vegetation is dominated by holm oak forests and maquis called ‘garriga’, the latter giving its name to the district. During the last few decades, this landscape has been transformed by agricultural activities, nowadays in recession. The main aim of this work was to collect and analyse the ethnoflora of this area in order to fill a gap in the ethnobotanical knowledge in Catalonia.

**Methods:**

The followed methodology was based on semi-structured interviews. The obtained data have been qualitatively and quantitatively analysed and compared with other available ones.

**Results:**

Data were gathered from 68 interviews involving 101 informants, whose ages range from 24 to 94, the mean being 73.07. The number of taxa reported in this study was 420, belonging to 99 botanical families. The interviewed informants referred 4715 use reports (UR) of 346 useful taxa, 1741 (36.93) of them corresponding to medicinal uses, 1705 (36.16%) to food uses, and 1269 (26.91%) to other uses. This study has inventoried, apart from individual plant uses, 260 plant mixtures, of which 98 are medicinal and 162 food. In the present study, 849 vernacular names with 116 phonetic variants have been collected, as well, for 410 taxa. The informant consensus factor (F_IC_) obtained for our interviewees is 0.93, and the ethnobotanicity index is 23.47% for the studied area. Apart from plants belonging to the typical Catalan, Iberian or European ethnofloras, the present work contributes information on some plants from semiarid or arid regions, such as *Artemisia herba-alba* and *Plantago albicans*, much rarer in the ethnobotany of the quoted areas.

**Conclusions:**

The results of this study reveal the persistence of ethnobotanical knowledge in the prospected area and the importance of filling the existing gaps in the ethnofloristic sampling of the Catalan territories. The almost complete dataset, now including some arid territories, will allow us to carry out a global analysis and to provide an accurate overview.

## Introduction

One hundred twenty-four years after ethnobotany’s first definition [1], Catalonia finds itself among the well-studied territories at the level of traditional knowledge on plant biodiversity [2]. Mountainous regions have been extensively sampled from the beginning of the practice of this science in our country [3–7], drawing an almost complete ethnobotanical map of the Catalan part of the Pyrenean mountain range [8]. Also profusely investigated is the coast and the two mountain ranges (littoral and prelittoral) that stretch along the coastline, from the area just north of Cap de Creus to Terres de l’Ebre in the south [9–17], basically constituting the Catalanidic territory from the physiographic point of view [18, 19], and plateaus and basins of central Catalonia, in the Auso-Segarric physiographic territory [20]. Apart from a few areas in the Pyrenees and the Ebro delta and its neighbouring territories, Catalan ethnobotanical prospection presents a significant gap in the Western plains. This area has a continental Mediterranean climate, with high thermic contrasts between warm and cold periods, and significantly dry, with almost five arid or perarid months per year [18, 21]. The soil is generally calcareous and, not rarely, saline or gypseous [22]. These territories, roughly coincidental with the Sicoric physiographic territory [19], are the only semiarid or arid lands in Catalonia, where some elements of steppe flora are present [23].

Although the arid zones have not attracted the interest of researchers as much as mountain or tropical areas, since, a priori, they are considered less rich and diverse compared to the zones with more exuberant vegetation, the arid Iberian lands have been studied in depth from the botanical point of view by the pioneering work of Bolòs [23] and Braun-Blanquet and Bolòs [24]. Some examples of botanical studies focused on flora and vegetation, carried out in areas close to the Garrigues district, may be found at Masclans [25], Boldú [26], Recasens and Conesa [27], Recasens et al. [28], Solé-Senan et al. [29], and the references contained in these papers. Concerning useful plants, the classical approach for arid lands has been the study of economic plants that could be grown or exploited in these areas for landscape protection and, mostly, agricultural and livestock raising activities (e.g. [30]). Ethnobotanical studies have also been carried out in arid and semiarid areas all around the world (e.g. [31–36]), including the Iberian Peninsula [37], although to date, none has yet been performed in Catalonia.

**Fig. 1:**
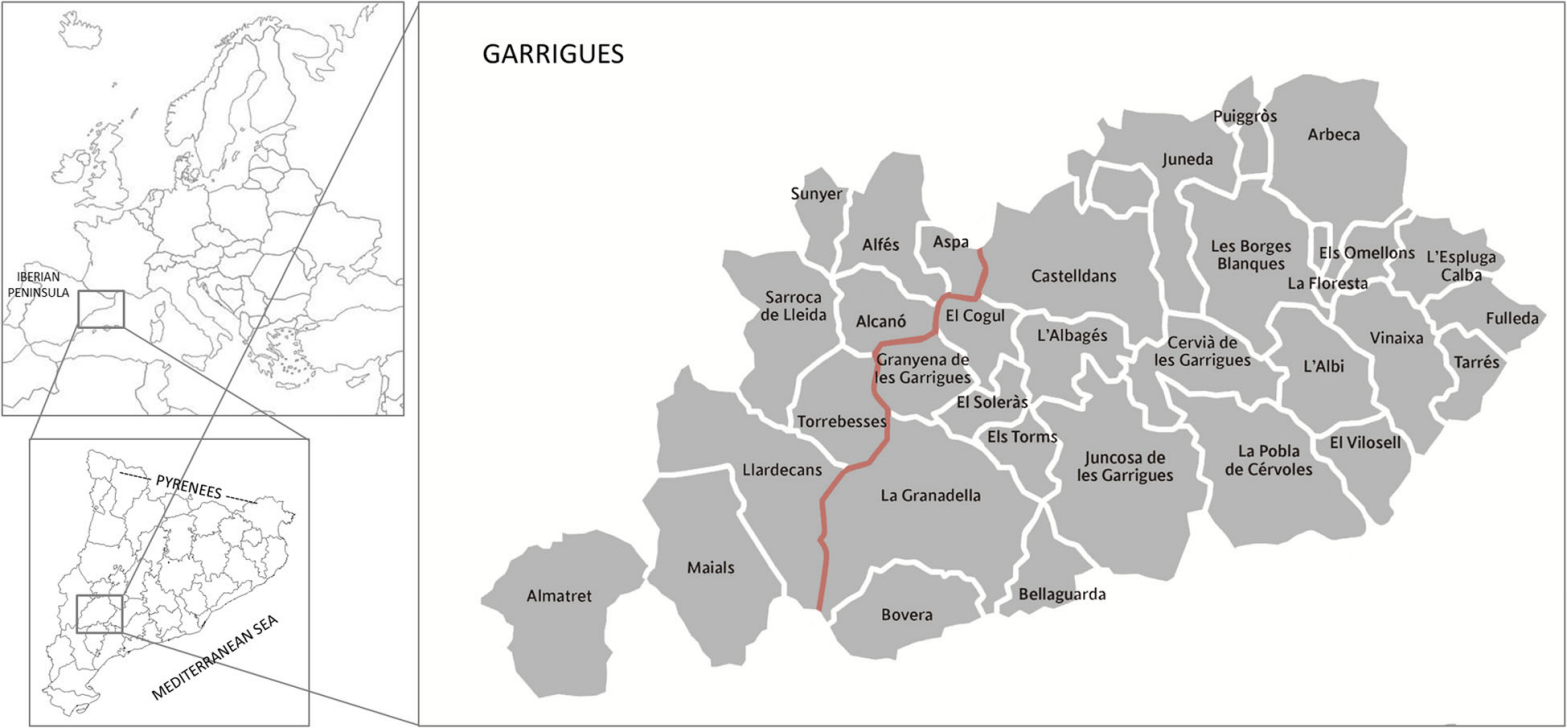
Map of the study area showing the location of Catalonia within Europe, the Garrigues district within Catalonia and the municipalities of the Garrigues *sensu lato.* The municipalities on the right of the red line correspond to the Garrigues *sensu stricto* and those on the left side, to historical Garrigues

The studied area, belonging to an arid region, is the Garrigues district (in Catalan ‘comarca’), located in west Catalonia, NE Iberian Peninsula (Fig. 1). Our study has focused on the Garrigues *sensu lato*, also including nine municipalities that had previously been located in the Garrigues district but currently belong to Segrià district and are known as historical Garrigues [38].

The Garrigues *sensu lato* has an altitude range from 201 m a.s.l., in the locality of Sarroca de Lleida, to 1021 m a.s.l., in Punta del Curull (Serra de la Llena, el Vilosell). The climate is low altitude continental Mediterranean, considered arid or semiarid, with a rainfall of about 333 mm/year. Winters are very cold and summers are very hot, with an annual mean of 14.4 °C with minimum and maximum temperatures of − 6.8 °C and 37.3 °C, respectively [39].

There are fundamental climax plant communities in the studied area. A kind of holm oak forest called ‘carrascar’ in Catalan language (*Quercetum rotundifoliae*) dominates a large part of the Garrigues district. A portion of the Garrigues, including the so-called historical (belonging to Segrià district) is more arid and dominated by a maquis (*Rhamno lycioidis-Quercetum cocciferae*) called ‘garriga’ (plural ‘garrigues’) in the Catalan language, which gives its name to the district. The part called Garrigues Altes (Serra de la Llena) is dominated by another type of holm oak tree forest (*Viburno tini-Quercetum ilicis*) called ‘alzinar’ in the Catalan language [40]. These climax communities are to a large extent degraded, and the landscape is far from the pure one of the plant formations quoted. Time and human action model a landscape fruit of the transformation of the natural vegetation by agricultural activities. In some cases, agriculture is currently in recession, and the abandoned fields will evolve again in terms of landscape.

The ‘carrascar’ forest degradation gives a ‘garriga’ poor in species and other formations such as *Rosmarino officinalis-Linetum suffruticosi*, *Genisto-Cistetum clusii*, *Ruto angustifolii-Brachypodietum retusi* or *Salsolo vermiculatae-Artemisietum herbae-albae*, this in part depending on the salinity and aridity of each area. In parallel, degradation of the maquis leads to the aforementioned communities plus *Delphinio gracilis-Lygeetum sparti*. Finally, ‘alzinar’ forest degradation gives rise to shrub or herbaceous formations such as *Erico multiflorae-Thymelaeetum tinctoriae* or *Phlomido angustifolii-Brachypodietum retusi* [40].

The study area is composed of 33 municipalities and comprises 1123.12 km^2^ and 22,243 inhabitants, representing a density of 19.80 inhabitants/km^2^ [41]. It is a very depopulated territory with an ageing population, 26.44% being over 65. The overageing index (quotient between the number of people aged 85 years and over and the number of people aged 65 and over, expressed as a percentage) is 21.59%, and the ageing index (quotient between the number of people aged 65 years and over and the number of young people under 15 years of age, expressed as a percentage) is 218.91% [41]. These data reflect a rather discouraging situation of the district in terms of population and highlight the importance of this study, since traditional biodiversity knowledge, in Western countries traditionally safeguarded to a considerable extent by old people, could fail to be transmitted, due to the lack or scarcity of young generations.

Economically, the Garrigues district can be defined as a rural area based on agriculture and livestock activities. Cereals, fruit and olive trees are the predominant crops, and poultry and pig farms, the main livestock. According to the agrarian register of 2009, the agricultural area occupies 60,745 ha and represents 54% of the district [41].

Between the second half of the eighteenth century and the end of the nineteenth century, a big agricultural expansion occurred in the studied area, coincidental with a significant increase in the population. This caused the brushwood clearing of difficult, abrupt zones covered by natural vegetation in order to become convenient for agricultural uses, originating a typical terrace structure. In 1865, the Urgell canal was opened, which brought water to northwest district municipalities (Arbeca, les Borges Blanques, Puiggròs, Juneda and, in part, Castelldans), allowing the diversification of crops: olive and almond trees, cereals in the dry farming lands, and alfalfa and maize in the irrigated lands [40]. Nowadays, the Urgell canal is not the only one, since, with the construction of the Segarra-Garrigues canal (2002–2030), the areas of possible irrigation are increasing, although the latter canal is not yet being used at 100% [42].

Even though western Catalonia arid lands have been the object of a relatively abundant number of botanical investigations, mostly centred on flora and vegetation [25–29, 43], the ethnobotanical background in the studied area is poor and could be described as practically inexistent. Just some ethnographic works have been done in the district, but not specifically focused on plants [44–46]. As for the neighbouring areas, only the Segarra district, not far from the Garrigues, but belonging to another physiographic territory, as above commented, has been studied from the ethnobotanical point of view [20].

The main aims of the present research are (i) to contribute towards filling a gap in the ethnobotanical knowledge map of Catalonia, (ii) to collect plant uses and their vernacular names in a depopulated arid or semiarid rural area, (iii) to analyse the ethnoflora in order to detect specific useful taxa of arid or semiarid areas and (iv) to establish some comparisons with other studied areas.

## Material and methods

### Fieldwork methods

The fieldwork method used was semi-structured interviews [47], always taking into account ethical principles of the International Society of Ethnobiology [48] and with the oral informed consent of the informants [49]. Interviews were carried out from May 2015 to July 2017 and were developed in the Catalan language, common to interviewers and interviewees. All information was digitalised, transcribed and introduced into our database (www.gestio.etnobotanica.cat) which contains all ethnobotanical data (on medicinal, food and other uses) collected by our research group.

The plant taxa cited by the informants were identified using the Flora Manual dels Països Catalans [22]. For systematic and nomenclatural aspects, we follow this quoted flora of the Catalan language area [22] for specific and infraspecific levels, and APG IV [50] for families. The herbarium vouchers have been deposited in the herbarium BCN (Centre de Documentació de Biodiversitat Vegetal, Universitat de Barcelona).

### Data analyses

The analyses were carried out with Excel (Microsoft Excel 2007) program. To analyse the results, we have used the use report (hereinafter, UR), i.e. the report of the use of one taxon by an informant, as the unit of measurement [51].

With the aim of assessing the state of ethnobotanical knowledge in the studied area, the following indices were calculated. The informant consensus factor (F_IC_) [52], which is the ratio of the number of UR minus the number of used taxa to the number of UR minus one, is more reliable when closer to 1. The ethnobotanicity index (EI) [53], which is the quotient between the number of plants used (only taking into account the native plants) and the total number of plants that constitute the flora of the territory, is expressed as a percentage. For this purpose, we have considered the plants present in the 10 × 10 km UTM squares corresponding to the studied area in the Banc de Dades de Biodiversitat de Catalunya [54]. The index of medicinal importance (MI) [55] was also calculated, dividing the total use reports for a specific use category by the number of taxa possessing this use.

We calculated the number of medicinal plants used per informant (MP/I), per inhabitant (MP/H), and per unit of area (MP/km^2^), in order to compare the results with other territories from which this information is provided only for this kind of useful plants.

For preparations with more than one plant, we obtained the recently proposed index of taxon usefulness in mixtures (ITUM) [56], which is the quotient between the number of reports of one taxon in mixtures and its total citations, whether simple or complex presentation. This index indicates the exclusiveness of taxa in mixtures when the value is one or closer to one.

Finally, for the vernacular names, we calculated the ethnophytonymy index [57], i.e. the percentage of taxa present in the flora that have folk names; the allochtonous ethnophytonymy index proposed by Carrió [58] assesses the rate between taxa having a vernacular name in non-Catalan languages (even for those taxa also having some Catalan names) and the total number of collected taxa; and the linguistic diversity index in phytonymy [59], i.e. the mean number of folk names for the plants recorded in the ethnofloristic prospection, computes the rate between the number of vernacular names and their taxa to evaluate the linguistic richness of a territory independently of its flora.

### Validation in the literature

To confirm the reported specific uses by participants in the study, we additionally reviewed the literature to carry out a pharmacological validation of most reported plants, those with three or more reports for a particular use, using monographs from official sources and encyclopaedic bibliography on phytotherapy [60–64].

## Results and discussion

### Characteristics of the interviewees

Data were gathered from 68 interviews to 101 informants. The interviewees’ ages range from 24 to 94, and the mean age is 73.07; 59.41% of them were, men and the remaining 40.59% were women, which is not common in Catalan ethnobotanical studies [6, 11, 13]. This deficit could be explained by the exodus of women to the cities, to work or to study, whereas men remained in the district. Nowadays, the Garrigues population has 1000 more men than women [41]. The informants were born in the studied area or have been living there a very significant part of their lives, and most of them are professionally related to agricultural and livestock raising activities.

### Plant taxa, botanical families and use reports

The number of taxa reported in this study was 420 (belonging to 99 botanical families), 25 of them have only been determined at the generic level and 46 presenting infraspecific category. In some cases, we are facing ethnotaxa, when all the species included in a genus are used indistinctly or when the informants were not able to distinguish them. The complete dataset of the recorded useful plants in the studied area is available in the Supplementary material.

The five best-represented families are Asteraceae (11.43%), Lamiaceae (7.38%), Fabaceae (7.14%), Rosaceae (5.71%) and Poaceae (5.00%). Two reasons could explain these results: on the one side, these families are large in terms of the number of species, and on the other, they are common in the Mediterranean landscape. We highlight the fifth place of the Poaceae in the UR ranking, insignificant in other ethnobotanical studies in Catalan regions. Some genera of this family are largely cultivated as cereal in the Garrigues and thus are well known and appreciated by local people. In addition, other genera belonging to the same family that grow wild there are typical of the arid and steppe regions, not only in the studied area. In agreement with the last assertion, this family occupies the first place in the ranking in an ethnobotanical prospection in a semiarid zone in Ethiopia [65].

The interviewed informants refer 4715 use reports (UR) of 346 useful taxa, 1741 (36.93) of them corresponding to medicinal uses, 1705 (36.16%) to food uses, and 1269 (26.91%) to other uses. The numbers show that a proportional sampling effort has been made for the three categories.

This first ethnobotanical approach to an arid or semiarid zone contributes 41 new taxa (9.76%) not mentioned before as useful in Catalonia (Table 1), one of them, *Moricandia arvensis* (L.) DC., being exclusive to the Sicoric physiographic territory [22]. In addition, among the 420 taxa mentioned, we find species with climatic and geographical affinities not common in previously studied territories: Mediterranean-Saharian connection species like *Atriplex halimus* L. or *Salsola vermiculata* L.; Iberian (European)-Pontian connection plants such as *Salsola soda* L.; Mediterraneo-Turanian connection taxa such as *Artemisia herba-alba* Asso or *Stipa parviflora* Desf.; Mediterranean plants of arid areas like *Lygeum spartum* L. or *Plantago albicans* L.; Iberian-Magrebian plants such as *Ononis tridentata* L. or *Retama sphaerocarpa* (L.) Boiss.; and typical Iberian endemics of these dry areas, such as *Dictamnus hispanicus* Webb ex Willk. [29].

**Table 1:** New contributions from the studied area to the ethnoflora of Catalonia

Taxon, family and herbarium voucher	Vernacular names (in Catalan if there is no another indication)	Medicinal use	Food use	Other use
*Antirrhinum barrelieri* Boreau subsp. *litigiosum* (Pau) O.Bolòs et Vigo (Plantaginaceae) BCN 150372	Esquitxagós, gallets, sabateta de la Mare de Déu			x
*Artemisia herba-alba* Asso (Asteraceae) BCN 129018	Botja, botja pudenta, espernallac bord, espernallac mascle	x		x
*Asphodelus cerasiferus* Gay (Asphodelaceae) BCN 125419	Albió, bironer	x		x
*Bupleurum fruticosum* L. (Apiaceae) BCN 156605	Matabou			
*Carlina corymbosa* L. subsp. *hispanica* (Lam.) O.Bolòs et J.Vigo (Asteraceae) BCN 125418	Assotacristos			x
*Centaurea paniculata* L. subsp. *leucophaea* (Jord.) Briq. (Asteraceae) BCN 140160	Trencacaps		x	
*Coronilla juncea* L. (Fabaceae) BCN 150377	Herba del cascat	x		
*Coronilla minima* L. subsp. *lotoides* (Koch) Nyman (Fabaceae) BCN 125401	Espernallac, herba del cascat	x		
*Coronilla valentina* L. subsp. *glauca* (L.) Batt. in Batt. et Trab. (Fabaceae) BCN 140154				x
*Crataegus azarolus* L. (Rosaceae) BCN 96759	Agret, atzeroler	x	x	
*Cynoglossum cheirifolium* L. (Boraginaceae) BCN 156570	Besneula	x		
*Cytinus hypocistis* (L.) L. (Cytinaceae) BCN 140178	Arnetes de mel, pinyetes, popetes, regineta, tenalletes de mel		x	x
*Ephedra distachya* L. (Ephedraceae) BCN 150358	Efedra	x		
*Helianthemum syriacum* (Jacq.) Dum.Cours. (Cistaceae) BCN 125478	Romer blanc	x		x
*Jasminum fruticans* L. (Oleaceae) BCN 125492	Gessamí			
*Kochia scoparia* (L.) Schrad. (Amaranthaceae) BCN 140172	Mirambell			x
*Lavatera maritima* Gouan (Malvaceae) BCN 14355	Malva marina	x		x
*Limodorum abortivum* (L.) Swartz (Orchidaceae) BCN 51622	Orquídia de color rosa			
*Limonium hibericum* Erben (Plumbaginaceae) BCN 96755				x
*Lonicera implexa* Ait. subsp. *implexa* (Caprifoliaceae) BCN 125512	Lligabosc	x		
*Moricandia arvensis* (L.) DC. (Brassicaceae) BCN 140180	Collejón (Spanish), espinac de frare		x	
*Oenothera biennis* L. (Onagraceae) BCN 81537	Onagra (Spanish)	x	x	x
*Oxalis pes-caprae* L. (Oxalidaceae) BCN 95553	Caramelets, vinagrera		x	x
*Peucedanum officinale* L. subsp. *stenocarpum* (Boiss. et Reut.) F.Q. (Apiaceae) BCN 99150	Cua de porc, fonoll de porc			
*Phlomis lychnitis* L. (Lamiaceae) BCN 156590	Candelera			
*Plantago albicans* L. (Plantaginaceae) BCN 125521	Herbafam	x	x	
*Pyrus spinosa* Forsk. (Rosaceae) BCN 103045	Saramenya (fruit), saramenyera		x	x
*Reseda luteola* L. (Resedaceae) BCN 100866	Gualda (Spanish), reseda (Spanish)			x
*Rhamnus lycioides* L. (Rhamnaceae) BCN 150374	Corniol	x	x	x
*Salix viminalis* L. (Salicaceae) BCN 65531	Vimen, vimenera	x		x
*Salsola kali* L. (Amaranthaceae) BCN 150378	Espantallops, parnella borda, trotamon	x		
*Salsola soda* L. (Amaranthaceae) BCN 42984	Parnella	x		x
*Salsola vermiculata* L. (Amaranthaceae) BCN 125398	Salada, siscall		x	x
*Scorzonera laciniata* L. (Asteraceae) BCN 125490	Barballa		x	
*Sideritis scordioides* L. (Lamiaceae) BCN 125397	Esparbonella, herba de Sant Antoni, planta de la pulmonia	x		
*Sideritis spinulosa* Barnades ex Asso (Lamiaceae) BCN 140155	Herba de la llanceta, herba de la pulmonia, herba del miracle	x		
*Stipa offneri* Breistr. (Poaceae) BCN 140524	Llambra			x
*Stipa parviflora* Desf. (Poaceae) BCN 125386	Bitxella, bitzell, pelaguer			x
*Tanacetum corymbosum* (L.) Schultz Bip. subsp. *corymbosum* (Asteraceae) BCN 125389	Herba de Santa Maria		x	
*Teucrium polium* L. subsp. *capitatum* (L.) Arcang. (Lamiaceae) BCN 140518	Timó mascle	x		
*Viscum album* L. subsp. *austriacum* (Wiesb.) Vollmann (Santalaceae) BCN 125518	Vesc, vesquera, visquercí, xoca	x	x	x

### General quantitative ethnobotany

With a view to assessing the state of ethnobotanical general knowledge in the studied area, we calculated the informant consensus factor (F_IC_) obtained for our interviewees (0.93), although we have also calculated the F_IC_ for medicinal information (0.89) in order to be able to compare it with other territories. It occupies a position in the upper part of the range of the values obtained for other Catalan language studied areas (Table 2).

**Table 2:** Quantitative ethnobotany indexes in other territories in Catalonia

Territory	F_IC_	EI	MP/informant	MP/inhabitant	MP/km^2^
Alt Empordà [13]	0.91	25.90	1.88	0.28 × 10^−2^	0.25
Cerdanya [3, 4]	0.93	_	1.11	0.82 × 10^−2^	0.23
*Garrigues* (this paper)	*0.89*	*23.47*	*1.94*	*0.88 × 10*^*−2*^	*0.17*
Gironès [17]	0.86	22.56	2.40	1.29 × 10^−2^	0.73
Montseny [11]	0.91	23.20	1.95	0.44 × 10^−2^	0.42
Pallars [5]	0.87	29.19	1.66	2.32 × 10^−2^	0.16
Ripollès [6]	0.96	28.60	1.73	1.10 × 10^−2^	0.29
Segarra [20]	–	–	3.17	0.54 × 10^−2^	0.13

F_IC_ informant consensus factor for medicinal information, EI ethnobotanicity index, MP number of medicinal plants

The ethnobotanicity index (EI), calculated not taking into account the 41 taxa of allochthonous plants recorded (those not present in Bolòs et al. [22]), is 23.47% for the studied area; this roughly means that between one fifth and one quarter of the plants of the area have been claimed as useful by the informants. The Garrigues EI is very close to Montseny and Gironès values, and slightly lower than in other Catalan language studied areas (Table 2).

### Medicinal uses

The total number of taxa employed for medicinal purpose was 196. Of the 1741 UR, 94.26% have been destined to human medicine, 4.02% to veterinary and 1.72% to both human and veterinary medicines (Supplementary material).

The number of medicinal plants used per informant is 1.94, value comprised between those found in Alt Empordà and Montseny (Table 2). A much higher value was obtained for the Segarra district, an area close to the Garrigues. However, this difference must be interpreted with caution, because it may be a bias partially due to the number of the informants interviewed (29 compared to 101). Medicinal plants per habitant are 0.88 × 10^−2^, a value similar to those recorded in Cerdanya or Ripollès (Table 2). Finally, medicinal plants per area are 0.17, a value near the one of Pallars, and lower than in other studied areas, like Montseny or Alt Empordà (Table 2).

*Thymus vulgaris* L. is the most cited taxon, followed by *Olea europaea* L. subsp. *europaea* var. *europaea*, and *Matricaria recutita* L. and *Ruta chalepensis* L. subsp. *angustifolia* (Pers.) Cout. The top twenty taxa and their main medicinal uses with three or more UR (according to the reliability criterion of Le Grand & Wondergem [66] and Johns et al. [67]) are shown in Table 3. Some of these taxa, either wild plants like *Thymus vulgaris* and *Sambucus nigra* L. or cultivated ones such as *Matricaria recutita* or *Allium sativum* L., are among the most cited in other Catalan territories [11, 13, 17].

**Table 3:** The top twenty taxa and their main medicinal uses with three or more UR

Taxon, family and herbarium voucher	Used part	Uses with UR ≥ 3	Pharmaceutical form	Total UR	Total UR (%)
*Thymus vulgaris* L. (Lamiaceae) BCN 96764	Aerial part	Anticatarrhal^1,2,5^; buccal anti-inflammatory; buccal antiseptic^3,5^; expectorant^1,2,3,5^; external antiseptic^3,5^; intestinal anti-inflammatory^2,5^; ocular antiseptic^5^. Pharyngeal anti-inflammatory^5^; salutiferous^3,5^; stomachic^2,3,5^; vulnerary^3,5^	Aerosol, bath, collutorium, gargarism, tisane	155	8.90
*Olea europaea* L. subsp. *europaea* var. *europaea* (Oleaceae) BCN 125505	Fruit, leaf	Analgesic, antihelminthic, antihypertensive^3,5^, antipyrotic, for earache, laxative^3^	Direct use, embrocation, liniment, tisane	89	5.11
*Matricaria recutita* L. (Asteraceae) BCN 140183	Inflorescence	Antibacterial^3,5^; antidismenorrhoeal^5^; digestive^2,3,4,5^; intestinal anti-inflammatory^1,2,3,4,5^; ocular antiseptic^5^	Bath, tisane	81	4.65
*Ruta chalepensis* L. subsp. *angustifolia* (Pers.) Cout. (Rutaceae) BCN 140153	Aerial part	Abortive^3,5,+^; antiasthmatic^5^; antineoplastic^5^; postpartum coadjuvant*; for sprains^5^; repellent	Direct use, tisane, unknown	81	4.65
*Santolina chamaecyparissus* L. subsp. *squarrosa* (DC.) Nyman (Asteraceae) BCN 96763	Aerial part	Antalgic/antiecchymotic/anti-inflammatory^3,5^; anti-edematous; anti-inflammatory^3,5^; anti-ulcerate^3^; buccal antiseptic^+^; cicatrizing; for sprains; vulnerary^+^	Bath, collutorium	69	3.96
*Jasonia saxatilis* (Lam.) Guss. (Asteraceae) BCN 125409	Aerial part	Anticatarrhal^3^; antihypertensive^3^; renal lithotriptic	Tisane	47	2.70
*Lippia triphylla* (L’Hér.) O. Kuntze (Verbenaceae) BCN 125394	Leaf	Antidismenorreic; intestinal anti-inflammatory^3^; digestive^3,5^; tranquilizer^3,5^	Tisane	37	2.13
*Malva sylvestris* L. (Malvaceae) BCN 125508	Aerial part, flower, leaf	Anticatarrhal^1,2^; antineoplastic; antitussive^1,2^; intestinal anti-inflammatory^1^; laxative; resolutive	Poultice, tisane, unknown	37	2.13
*Vitis vinifera* L. (Vitaceae) BCN 150353	Fruit	Anti-inflammatory/muscular pain; pharyngeal anti-inflammatory; for sprains	Bath, direct use, poultice	36	2.07
*Rosmarinus officinalis* L. (Lamiaceae) BCN 156603	Aerial part	Anticatarrhal^5^; antihypertensive	Tisane	33	1.90
*Allium sativum* L. (Amaryllidaceae) BCN 29832	Bulb	Against bee bites^5^; analgesic^5^; antibacterian^3,5^; antihelminthic^3,5^; for earache^5^; for warts^5^; salutiferous^3,5^	Direct use, poultice	32	1.84
*Sambucus nigra* L. (Adoxaceae) BCN 96771	Inflorescence	Anticatarrhal^1,2,5^; ocular antiseptic	Bath, tisane	30	1.72
*Juglans regia* L. (Juglandaceae) BCN 150364	Leaf	Cicatrizing^3^; resolutive	Bath	28	1.61
*Dictamnus hispanicus* Webb ex Willk. (Rutaceae) BCN 125519	Aerial part, leaf	Abortive; antihypertensive	Tisane	27	1.55
*Eryngium campestre* L. (Apiaceae) BCN 125407	Aerial part	Antiophidian; for rash	Direct use	27	1.55
*Equisetum ramosissimum* Desf. (Equisetaceae) BCN 29982	Aerial part	Antihypertensive; diuretic	Tisane	26	1.49
*Euphorbia* sp. (Euphorbiaceae)	Latex	For warts	Direct use	25	1.44
*Salvia officinalis* L. subsp. *lavandulifolia* (Vahl) Gams (Lamiaceae) BCN 96762	Leaf	Antihypertensive^5^; hematocathartic	Tisane	25	1.44
*Hypericum perforatum* L. (Clusiaceae) BCN 96760	Flower	Antalgic/antiecchymotic/anti-inflammatory^4,5^; for skin disorders	Liniment	24	1.38
*Satureja fruticosa* (L.) Briq. subsp. *fruticosa* (Lamiaceae) BCN 125387	Aerial part	Digestive^3,5^; intestinal anti-inflammatory^3,5^	Tisane	24	1.38

The number of UR is the total number, not only the UR according to uses with three or more use reports. Uses with no sign are addressed only to humans, those marked with an asterisk (*) only to animals, and those with a plus sign (+) are common to both. Validation uses: ^1^EMA [62], ^2^ESCOP [63], ^3^Fitoterapia.net [64], ^4^Blumenthal [60], and ^5^Duke [61]

Conversely, other species like *Jasonia saxatilis* (Lam.) Guss., *Santolina chamaecyparissus* L. subsp. *squarrosa* (DC.) Nyman or *Dictamnus hispanicus* Webb ex Willk., commonly growing and frequently reported in the present studied area, are not the most common in top positions in other territories, where they are less abundant or not present. This fact reflects the well-confirmed idea that the closer to civilization a plant grows, the more it is used by local people [67].

The medicinal taxa recorded belong to 68 families. In this category, Lamiaceae is the most reported with 18.78%, followed by Asteraceae (17.06%) and, far from them, Rutaceae (6.49%). These results are close to those recorded in other ethnofloristic works conducted in Mediterranean areas, where predominating families Asteraceae, Lamiaceae and Rosaceae occupy first places [68]. The relevant presence of Rutaceae has its explanation in the specific use of their main species reported, *Ruta chalepensis* subsp. *angustifolia* for abortive use (in both animals and humans) and postpartum coadjuvant (in animals) and *Dictamnus hispanicus* for antihypertensive use.

Plant parts most commonly used for remedies preparation are the aerial part with 45.78% including young, sterile, flowering, and fructified aerial parts; these organs are followed by leaves (17%). These results agree with those in other Catalan language areas such as Segarra [20] and Pallars [5], the first one closer and not very different in the climate to the Garrigues and the second one farther and with mountain (including high mountain) conditions.

**Fig. 2:**
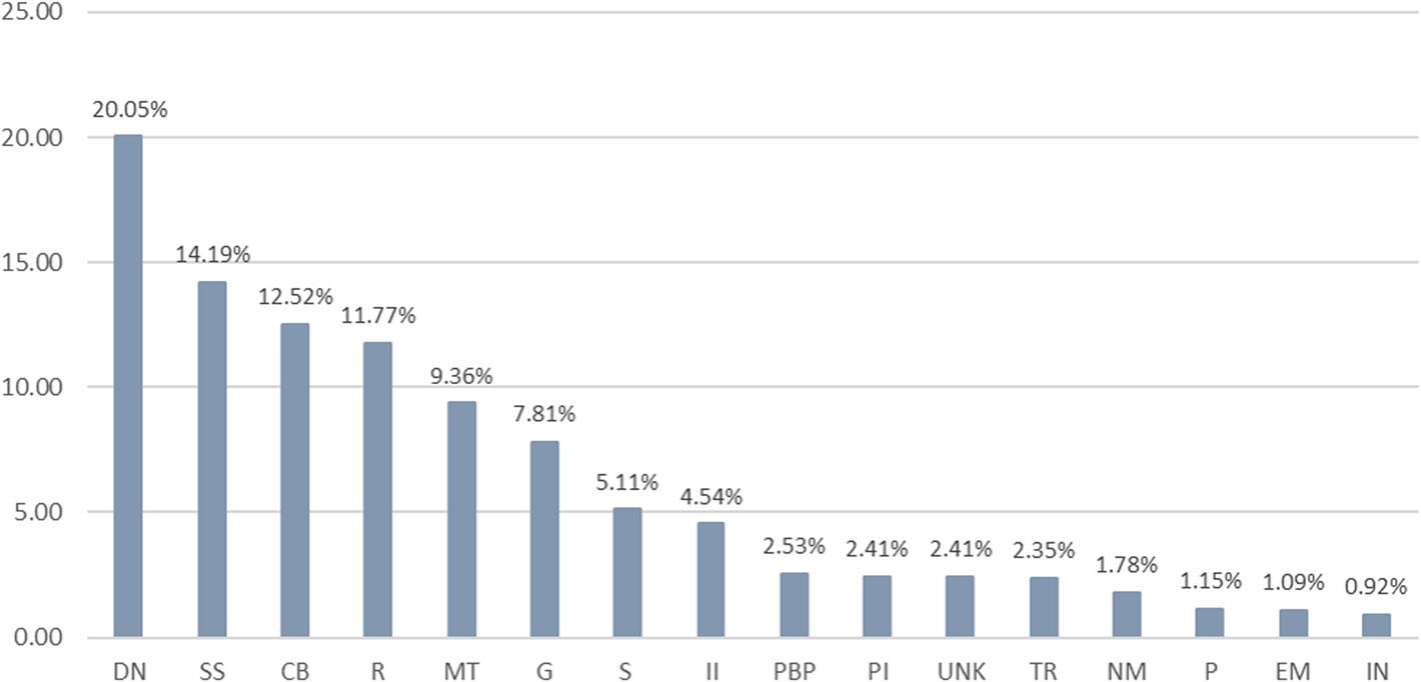
Systems and disorders addressed with medicinal plants in the area studied. Abbreviations: DN, digestive system and nutritional disorders; SS, skin and subcutaneous tissue disorders; CB, circulatory system and blood disorders; R, respiratory system disorders; MT, musculoskeletal system disorders and traumas; G, genitourinary system disorders; S, sensory system disorders; II, infections and infestations; PBP, pregnancy, birth, and puerperal disorders; PI, pain and inflammations; UNK, unknown; TR, tonic and restorative; NM, nervous system and mental disorders; P, poisoning; EM, endocrine system and metabolic disorders; IN, immune system disorders and neoplasia

The most treated troubles, representing 58.53% of the total, are addressed to digestive system and nutritional disorders, skin and subcutaneous tissue disorders, circulatory system and blood disorders and respiratory system disorders (Fig. 2). If we analyse the reports for specific medicinal use, antihypertensive (8.10%), anticatharral (7.01%), intestinal anti-inflammatory (4.77%), antialgic/anti-ecchymotic/anti-inflammatory (3.79%) and ocular antiseptic (3.73%) are the most reported.

Thirty plant taxa have been reported as antihypertensive in the Garrigues district, occupying the first position among the most cited plants for specific disorders. The plants used to lower blood pressure are commonly quoted in other ethnobotanical studies in Catalonia, but in no one case they occupy the first position [3–6, 11, 13].

**Fig. 3:**
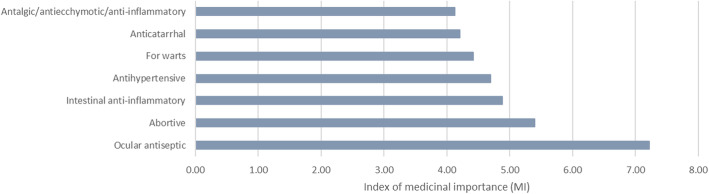
The seven medicinal specific categories with an index of medicinal importance (MI) higher than 4

The index of medicinal importance is useful to evaluate the real importance of the use, as a specific use can be cited for a few or many species and this may change the relevance of the information. This index was calculated for 20 of 134 medicinal use categories (those most reported), and the results for the uses that have a ratio higher than 4 are shown in Fig. 3.

Regarding the pharmaceutical form, tisane, including decoction and infusion, represents 39.06% of the total forms reported, followed by bath (16.03%) and direct use (internal or external) (14.65%). Among the thirty pharmaceutical forms employed, the simplest ones predominate over others; other more complex ones, such as poultice or essence, are also used but in low frequency.

#### Pharmacological validation

To confirm the top twenty taxa (excepting *Euphorbia*, only determined at the genus level) and their main medicinal uses with three or more use reports, we reviewed the literature (Table 3). Seventy-seven uses of these 19 taxa are coincidental with those recorded in the literature set consulted. For an important percentage, 59.74%, of the ethnobotanical uses, we validated the information with at least one of the sources checked. By far, Duke’s CRC handbook of medicinal herbs [61] with 48.05% of validated uses and Fitoterapia.net [62] with 33.77% were the most inclusive, systematic and detailed works analysed. The traditional uses confirmed in this literature, which is often used in drug register processes, may be considered as the most consolidated, with a view to introducing new products onto the market. Whereas the roughly 40% of the ethnobotanical uses (all of them reliable in terms of the number of three or more use reports [66, 67]) not found in the literature set should be the object of further phytochemical and/or pharmacological investigation.

### Food uses

In the studied region, 168 taxa consumed as food were detected, 52.98% of them being cultivated and the remaining 47.02% wild. Of the 1705 use reports, 91.79% have been consumed by humans, 8.03% by animals and 0.18% by both humans and animals (Supplementary material). The number of food plants cited per informant is 1.66.

The five most cited species are *Thymus vulgaris*, *Papaver rhoeas* L., *Rubus ulmifolius* Schott, *Olea europaea* subsp. *europaea* var. *europaea* and *Prunus dulcis* (Mill.) Weeb. The first three plants are wild and typical in the studied area: The aerial part of *Thymus vulgaris* is used as a condiment, the young leaves of *Papaver rhoeas* consumed as salad and the fruit of *Rubus ulmifolius* is either eaten fresh or cooked with sugar, to make jam, the three uses being quite common. *Olea europaea* subsp. *europaea* var. *europaea* and *Prunus dulcis* are the main crops in the studied area, where they are largely cultivated (ca. 19,276 and 645 ha, respectively [41]), and this explains their high number of use reports. The fundamental role of oil in the Mediterranean cuisine together with the numerous recipes for the preparation and preservation of olives explain the high number of citations of the olive tree. The high number of UR of the almond tree could be interpreted in the same sense. This species, widely distributed in the Mediterranean area, has been cited for the consumption of its ripe seeds, raw or toasted, but also for the complete unripe fruit, named green almonds, which are usually eaten either directly or pickled. The top-ranked twenty species and their principal mode of consumption are shown in Table 4.

**Table 4:** The top twenty taxa and their main food uses with three or more UR

Taxon, family and herbarium voucher	Used part	Uses with UR ≥ 3	Total UR	Total UR (%)
*Thymus vulgaris* L. (Lamiaceae) BCN 96764	Aerial part	Boiled in water, condiment	74	4.34
*Papaver rhoeas* L. (Papaveraceae) BCN 125507	Aerial part, leaf	Raw	69	4.05
*Rubus ulmifolius* Schott (Rosaceae) BCN 156557	Fruit	Cooked with sugar, high-grade alcoholic beverage, raw	69	4.05
*Olea europaea* L. subsp. *europaea* var. *europaea* (Oleaceae) BCN 125505	Fruit, leaf	Air-dried*, macerated in water, preserved in oil, preserved in vinegar	65	3.81
*Prunus dulcis* (Mill.) Weeb. (Rosaceae) BCN 125495	Fruit, seed	Air-dried, cooked with oil, cooked with sugar, milk-based beverage, preserved in vinegar, raw, toasted, water-based beverage	65	3.81
*Satureja montana* L. (Lamiaceae) BCN 125403	Aerial part	Condiment	56	3.28
*Sonchus oleraceus* L. (Asteraceae) BCN 125509	Leaf	Raw	52	3.05
*Arbutus unedo* L. (Ericaceae) BCN 96768	Fruit	Cooked with sugar, raw	50	2.93
*Ficus carica* L. (Moraceae) BCN 150361	Fruit	Air-dried, high-grade alcoholic beverage, raw	49	2.87
*Celtis australis* L. (Cannabaceae) BCN 125477	Fruit	Raw	48	2.82
*Rosmarinus officinalis* L. (Lamiaceae) BCN 156603	Aerial part	Condiment	45	2.64
*Cydonia oblonga* Mill. (Rosaceae) BCN 150356	Fruit	Cooked with sugar	38	2.23
*Sorbus domestica* L. (Rosaceae) BCN 150384	Fruit	Air-dried, raw	37	2.17
*Foeniculum vulgare* Mill. subsp. *piperitum* (Ucria) Cout. (Apiaceae) BCN 125404	Aerial part, fruit	Boiled in water, condiment, raw	34	1.99
*Malva sylvestris* L. (Malvaceae) BCN 125508	Flower, fruit	Raw	34	1.99
*Beta vulgaris* L. subsp. *maritima* (L.) Arcang. (Amaranthaceae) BCN 156567	Leaf	Boiled in water, raw	33	1.94
*Silybum marianum* (L.) Gaertn. (Asteraceae) BCN 125516	Inflorescence	Raw	33	1.94
*Silene vulgaris* (Moench) Garcke (Caryophyllaceae) BCN 96770	Leaf	Boiled in water, cooked with oil, raw	31	1.82
*Solanum lycopersicum* L. (Solanaceae) BCN 29952	Fruit	Cooked with sugar, preserved	31	1.82
*Laurus nobilis* L. (Lauraceae) BCN 150355	Leaf	Condiment	30	1.76

The number of UR is the total number, not only the UR according to uses with three or more use reports. Uses with no sign are for humans and those marked with an asterisk (*) are for animals

The most consumed parts of these plants are the fruits or infructescences, with 47.98% of the total, and, significantly lower, aerial parts (22.35%) and leaves (18.83%). The explanation is the extensive use of olives and almonds, but also of wild or marginal fruits such as *Rubus ulmifolius*, *Celtis australis* L., *Pyrus spinosa* Forsk., *Sorbus domestica* L., *Crataegus azarolus* L. and *C. monogyna* Jacq. Fruits are popular for consumption around the world. To quote similar climatic areas to the one considered, in arid zones of India, the relevance is remarked of local fruits in people’s nutrition, confirmed by composition analyses, even though they are somewhat undervalued and underutilised when compared with commercial exotic fruits [69].

The most common mode of consumption is fresh (43.11%) and as a condiment (16.07%). Fresh consumption includes raw salads like *Eruca vesicaria* (L.) Cav. subsp. *sativa (Mill.) Thell. in Hegi*, *Papaver rhoeas*, *Sonchus oleraceus* L., *Samolus valerandi* L., the above-mentioned edible fruits or more uncommon products like the fresh inflorescence of *Silybum marianum* (L.) Gaertn. This last mentioned plant is not so frequently eaten, but its preparation and consumption are comparable to that of another plant of the same family, *Carlina acanthifolia* All., very popular in mountain regions*.* Condiments are mostly used to flavour different types of cooked meat and snails, among others, and, as we have mentioned previously, to preserve, and also to flavour, the olives.

### Medicinal and food mixtures

This study has inventoried, apart from individual plant uses, 260 plant mixtures, of which 98 (37.69%) are medicinal and 162 (62.31%) food (Supplementary material).

Sixty-one taxa are employed in medicinal mixtures, and the mean of species reported for mixture is 2.67. The most reported species are *Thymus vulgaris*, present in 10.69% of mixtures; *Olea europaea* subsp. *europaea* var. *europaea* (6.87%); and *Pinus halepensis* Mill. (5.34%).

The index of taxon usefulness in mixtures was calculated taking into account all the taxa with three or more use reports in mixtures, thereby using the same criteria as in the simple presentation [66, 67]. The results in medicinal mixtures show there are two taxa reported only in mixtures, *Petroselinum crispum* (Mill.) Hill and *Apium graveolens* L., with the maximum ITUM value of one, and another one, *Citrus limon* (L.) Burm., with a high value (0.92).

These medicinal mixtures are used to treat, principally, respiratory disorders (29.59%), circulatory system and blood disorders (17.35%) and immune system disorders (11.22%). The most common pharmaceutical form does not change in respect to individual plants, tisane being reported in half of the mixtures, followed by poultice (14.29%). The informant consensus factor (F_IC_) for medicinal mixtures data is 0.77, a value between Gironès district (0.56) [17] and the study carried out in two Catalan territories (Alt Empordà and Ripollès) (0.85) [56].

In food mixtures, 78 taxa are reported. The mean number of species reported for food mixtures is 3.59, and the most cited species are *Allium sativum* (5.16%), *Thymus vulgaris* (4.65%), *Juglans regia* L. and *Satureja montana* L. (4.48%, for both).

The number of taxa only reported in food mixtures is higher than the number of taxa in the medicinal ones. The analysis carried out shows seven taxa with a maximum value (ITUM = 1), indicating their use only in mixtures. Most of them are spices like *Cinnamomum verum* J.Presl, *Coriandrum sativum* L., *Illicium verum* Hook.f., *Myristica fragrans* Houtt., *Piper nigrum* L. or *Syzygium aromaticum* (L.) Merr. et Perry, but also *Hyssopus officinalis* L. These high ITUM values could be explained by the fact that all of these taxa are used as a seasoning and they are rarely used alone.

Condiment is the main use reported (45.06%), followed by high-grade alcoholic beverage (12.35%). Analysing in more details the condiment use, we have counted 43 recipes regarding seasoning olives, and this means 26.54% of the total of the mixtures. The relevant percentage of high-grade alcoholic beverage uses is mostly due to the importance of the traditional liqueur called *ratafia*. Finally, the informant consensus factor (F_IC_) for food mixtures is 0.87. Even if this value is not comparable with other studies, because to date, scarce attention has been paid to food mixtures in comparison with medicinal ones it suggests, its maximum value being 1, a solid information fact of popular knowledge on plant combinations with food use.

### Other uses

This category includes non-medicinal and non-food uses. Nowadays, these uses are the most vulnerable. On the one hand, the application of this melting pot with numerous use subcategories seems not as necessary as in the past, and the traditional work has been mechanised, or many objects for home use are currently acquired in supermarkets. On the other hand, unlike plants with medicinal and food uses, traditional knowledge of plants with other uses has not been much investigated. Serving as good examples, pharmaceutical companies are interested in the chemical compounds of some of the plants traditionally used as medicine [70], and leading chefs are looking for new ingredients to incorporate new flavours and textures in their dishes and wild food plants [71]; conversely, domestic uses and handicraft products are minimal at a general level in business terms.

Nevertheless, although most of them have almost disappeared and others remain in a residual condition, specific research to analyse this kind of uses is still important. Not least, they are highly considered by their practitioners and have a cultural relevance and may even imply some economic revenues to local people [72] or generate new market opportunities in a world where people are increasingly concerned about the environment and try to reduce plastics and other waste.

In our study, we have collected 1269 UR concerning 184 taxa (Supplementary material). The number of plants cited for other uses (as stated, all but medicinal and food) per informant is 1.82. Table 5 shows the top twenty species in this field and their principal uses (with three or more use reports). The five most reported species are *Arundo donax* L., mostly indicated for their use in home gardens and, in general, in agricultural management; *Olea europaea* subsp. *europaea* var. *europaea*, to elaborate handmade soap from the used oil or to bring a piece of aerial part of the olive tree to bless during Palm Sunday; *Celtis australis* for the elaboration of forks; *Dorycnium pentaphyllum* Scop. for broom elaboration; and *Helichrysum stoechas* (L.) Moench for decorative use.

**Table 5:** The top twenty taxa and their main other uses with three or more UR

Taxon, family and herbarium voucher	Used part	Uses with UR ≥ 3	Total UR	Total UR (%)
*Arundo donax* L. (Poaceae) BCN 156562	Inflorescence, stem, whole plant	Agrosilvopastoral management, basketry, domestic, ludic, unclassified	79	6.23
*Olea europaea* L. subsp. *europaea* var. *europaea* (Oleaceae) BCN 125505	Aerial part, ash, fruit, stem	Cane elaboration, domestic, fuel obtention, magic and religious beliefs and practices	75	5.91
*Celtis australis* L. (Cannabaceae) BCN 125477	Seed, stem	Agrosilvopastoral management, cane elaboration, ludic	72	5.67
*Dorycnium pentaphyllum* Scop. (Fabaceae) BCN 125504	Aerial part, flower	Broom elaboration, honey obtention	52	4.10
*Helichrysum stoechas* (L.) Moench (Asteraceae) BCN 96758	Aerial part, inflorescence	Air freshener, bouquet elaboration, broom elaboration, honey obtention, magic and religious beliefs and practices	49	3.86
*Genista scorpius* (L.) DC. in Lam. et DC. (Fabaceae) BCN 156592	Aerial part	Domestic, for pig slaughter, fuel obtention, unclassified	47	3.70
*Mantisalca salmantica* (L.) Briq. et Cavill. (Asteraceae) BCN 125420	Aerial part	Broom elaboration	34	2.68
*Typha latifolia* L. (Typhaceae) BCN 31314	Aerial part, leaf	Agrosilvopastoral management, bouquet elaboration, unclassified	33	2.60
*Viscum album* L. subsp. *austriacum* (Wiesb.) Vollm. (Santalaceae) BCN 125518	Aerial part, stem	For hunting, magic and religious beliefs and practices, recollection for selling	32	2.52
*Prunus dulcis* (Mill.) Weeb. (Rosaceae) BCN 125495	Ash, flower, stem	Domestic, fuel obtaining, honey obtention	31	2.44
*Lygeum spartum* L. (Poaceae) BCN 125497	Aerial part	Agrosilvopastoral management, rope elaboration, shoe elaboration	28	2.21
*Lavandula latifolia* Medic. (Lamiaceae) BCN 96766	Aerial part, whole plant	Air freshener, bouquet elaboration, cosmetic, for gardening	24	1.89
*Pinus halepensis* Mill. (Pinaceae) BCN 150381	Ash, stem, whole plant	Domestic, folk oral literature, fuel obtention	24	1.89
*Rosmarinus officinalis* L. (Lamiaceae) BCN 156603	Aerial part, flower	Cosmetic, smoking, honey obtention	24	1.89
*Sorbus domestica* L. (Rosaceae) BCN 150384	Fruit, stem, whole plant	Folk oral literature, unclassified	23	1.81
*Urtica urens* L. (Urticaceae) BCN 150351	Aerial part	Agrosilvopastoral management	22	1.73
*Linum tenuifolium* L. subsp. *suffruticosum* (L.) Litard. (Linaceae) BCN 125496	Whole plant	Folk oral literature	20	1.58
*Quercus coccifera* L. (Fagaceae) BCN 150369	Aerial part, stem	Domestic, cane elaboration, fuel obtention	20	1.58
*Pistacia lentiscus* L. (Anacardiaceae) BCN 125514	Aerial part, fruit	For hunting, repellent	18	1.42
*Aphyllanthes monspeliensis* L. (Asparagaceae) BCN 125510	Root, whole plant	Domestic, recollection for selling, unclassified	16	1.26

The number of UR is the total number, not only the UR according to uses with three or more use reports

The aerial part and stems represent almost a third part of all reports. The first one, the most cited, is represented by 50.83%, and the second one, almost by a quarter (21.99%).

**Fig. 4:**
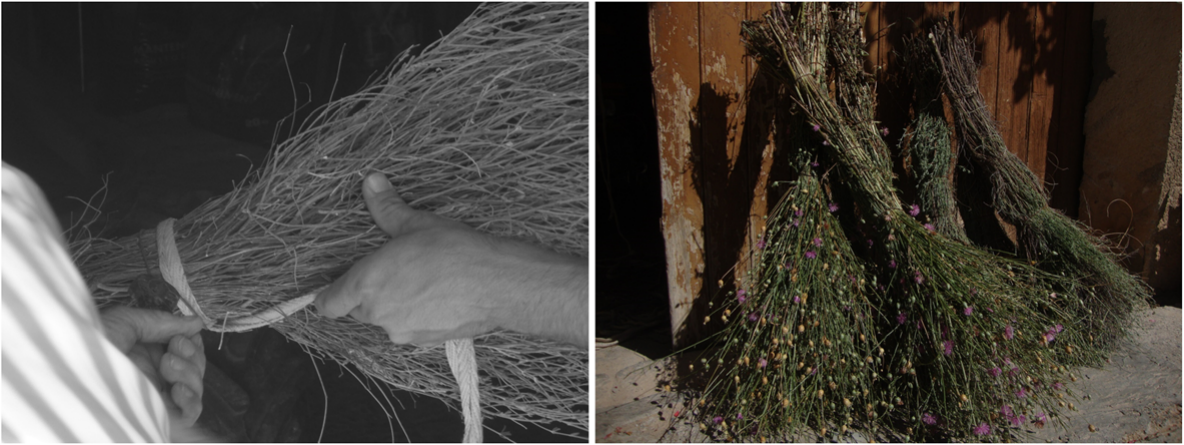
A moment of broom elaboration process with *Dorycnium pentaphyllum* Scop. and some freshly produced brooms of this species and *Mantisalca salmantica* (L.) Briq. et Cavill.

If we split data into subcategories, agrosilvopastoral management (11.74%), domestic (8.67%), magic or religious beliefs and practices (8.35%) and broom elaboration (8.20%) are the most reported. The first three categories are general and group a variety of uses, which explains the first positions. Broom elaboration is a very specific use, quoted by almost all our informants and a wide diversity of taxa. Up to 11 species are reported for this use. *Dorycnium pentaphyllum* and *Mantisalca salmantica* (L.) Briq. et Cavill. are the most valued, specifically used to sweep the threshing floor after selecting cereal or legume grains, and the street near home (Fig. 4). Other species reported are *Arundo donax*, *Chamaerops humilis* L., *Erica multiflora* L., *Kochia scoparia* (L.) Schrad., *Lygeum spartum*, *Phoenix dactylifera* L., *Retama sphaerocarpa*, *Rosmarinus officinalis* L. and *Spartium junceum* L. Some of these brooms continue, to some extent, to be used nowadays and renewed when necessary. Furthermore, even if, as already stated, these uses are, in general, declining, some utilisations, such as ritual (for instance in Christmas or Palm Sunday), folk oral literature (e.g. proverbs) or handicraft basketry, persist in the studied territory, as they do in other Catalan language territories [72] and in other Mediterranean areas [73, 74].

### Vernacular names

In the present study, 849 vernacular names with 116 phonetic variants have been collected for 410 taxa. Fifty-four of these taxa do not have medicinal, food or other uses associated, 36 of them with other observations and 18 only with a popular name. Ten taxa have been mentioned by the informant without any popular name; in these few cases, the informant did not know or could not remember the name of a plant. Out of the 849 names, 79 are in the Spanish language, two in French and one in English, all the rest being in Catalan. The most cited taxon in terms of folk name (apart from cultivated plants with races, see later) is *Thymus vulgaris*, which has been reported 90 times with the popular name *timó* and 17 as *farigola*, the second name (belonging to the so-called eastern Catalan dialect) not typical in the area (speaking western Catalan dialect), but known by many informants as used in other Catalan language variants. The same happens with *Rosmarinus officinalis*’ names, with ‘romer’ (75 UR) as the typical in the district and ‘romaní’ (5 UR) also known from other dialects.

The ethnophytonymy index for vernacular names shows a value close to the classical ethnobotany index 21.80%; this indicating that most of the plants have at least one vernacular name in the Catalan language.

The allochthonous ethnophytonymy index is 18.05%, between Gironès (4.7%) [17] and Mallorca (27.8%) [58] values. The reason for this quite high value in a rural district is the origin of one of the interviewees, who is from Germany but has lived in the Garrigues district for a long time, although she only speaks Spanish, not Catalan.

The linguistic diversity index in phytonymy, calculated to value the linguistic richness of a territory independently of its flora, reaches a value of 1.98 for taxa with only Catalan language names and 2.07 for all taxa.

Finally, we want to mention the interesting case of a certain number of plant names that have gender adjectives although they are not dioecious taxa. Some plants are named based on other taxa’s names with the addition of ‘mascle’ (male) or ‘femella’ (female), irrespective of their sexual condition. As already stated, ‘timó’ is the typical name for *Thymus vulgaris*; based on this, *Teucrium* gr. *polium* (belonging to the same family as *Thymus*) and *Coris monspeliensis* subsp. *monspeliensis* (from another family) are termed ‘timó mascle’. *Santolina chamaecyparissus* subsp. *squarrosa* is named ‘espernallac’ or ‘espernallac femella’, whereas *Artemisia-herba-alba* is known as ‘espernallac mascle’; in this case, people commenting that the former species produces flowers and the latter does not (in fact, *Artemisia* capitula and flowers are much smaller and less visible than those of *Santolina*).

The Garrigues being an important agricultural area, it is not surprising that some cultivated plants bear a high number of names due to the races present in the area or known by the informants. This is the reason for which *Vitis vinifera* L. (with 15 folk names) is the taxon with the highest number of popular names. This also happens with *Olea europaea* subsp. *europaea* var. *europaea* (13 names), with the added value, at a local level, that one of the villages of the district (Arbeca) gives the name to a very well-known olive race (‘arbequina’), highly appreciated for both fruit consumption and oil elaboration [75]. Finally, the same applies to *Triticum aestivum* L. (14 names). The first wild plant species in the ranking of common names is *Helichrysum stoechas* (11 names), followed by *Crataegus monogyna* and *Silybum marianum* (both with nine names).

## Conclusions

This first study in the Western plains has revealed the importance of prospecting the arid or semiarid areas of Catalonia. Traditional knowledge is persisting in the district, as the results confirm, in terms of high values of the number of useful (medicinal, food or with other applications) taxa, number of popular phytonyms, ethnobotanicity index and informant consensus factor. The persistence of traditional knowledge is comparable to that existing in other Catalan areas regarding medicinal, food and even other plant uses (e.g. [8, 17, 72]), which is of interest to confirm in this first study focused on an arid land. Indeed, most ethnofloristic parameters fall within the general corpus of Catalan, Iberian and European ethnobotany, but some particularities arise. The climatic and soil characters, together with the agricultural condition of the territory, give rise to the Poaceae among the five top families recorded in the number of taxa known and used. The characteristics of the territory bring considerable relevance to plants with semiarid or arid, and steppe, affinities, which are rarely reported in other places of the above-quoted areas. This is why this paper contributes 41 novelties of taxa to the Catalan ethnoflora, irrespective of it having been quite profusely studied. With the present work, the ethnofloristic gap in the arid Catalan lands is at least partly filled. Apart from the fact that some specific prospections are still needed, this may be seen as the first step towards starting work with all available ethnobotanical data in the country in order to perform meta-analytic studies. In its turn, these studies should provide the basis for comparisons at higher levels, such as Iberian and Mediterranean.

## Supplementary information

**Additional file 1.** Supplementary information.

## Data Availability

All data are available in the Supplementary information.
